# The developmental trends of parental self-efficacy and adolescents’ rule-breaking behaviors in the Italian context: A 7-wave latent growth curve study

**DOI:** 10.1371/journal.pone.0293911

**Published:** 2023-11-15

**Authors:** Chiara Remondi, Maria Gerbino, Antonio Zuffianò, Concetta Pastorelli, Eriona Thartori, Dario Bacchini, Laura Di Giunta, Carolina Lunetti, Ainzara Favini, Jennifer E. Lansford, Kenneth A. Dodge

**Affiliations:** 1 Department of Psychology, Sapienza University of Rome, Rome, Italy; 2 Department of Human Studies, University of Naples “Federico II”, Naples, Italy; 3 Faculty of Educational Sciences, Guglielmo Marconi University, Rome, Italy; 4 Center for Child and Family Policy, Duke University, Durham, North Carolina, United States of America; Fondazione Policlinico Universitario Agostino Gemelli IRCCS, Universita’ Cattolica del Sacro Cuore, ITALY

## Abstract

Parental self-efficacy (PSE) captures parents’ beliefs in their ability to perform the parenting role successfully and to handle pivotal issues of specific developmental periods. Although previous studies have shown that, across the transition to adolescence, parents show decreasing levels of PSE while adolescents exhibit increasing engagement in rule-breaking (RB) behaviors, there is a paucity of studies investigating whether and how changes in PSE are related to late adolescents’ RB behaviors across development. The present study examined the developmental trends of PSE among Italian mothers and fathers over seven waves (representing children’s transition from late childhood to late adolescence; approximately from 9 to 18 years old) as well as the longitudinal associations between PSE and RB behaviors during late adolescence. Data were drawn from seven waves of the Parenting Across Cultures (PAC) project, a large-scale longitudinal, cross-cultural study, and included 200 Italian children (*M*_AgeAtTime1_ = 9.80, *SD* = 0.65; 50.5% girls) and their parents (200 mothers; 190 fathers). PSE was measured across all seven time-points (from T1 to T7), while adolescents’ RB behaviors were measured at the first and last assessment (T1 and T7). Results of univariate latent growth models showed a cubic trend of mothers’ PSE, which revealed a decreasing pattern characterized initially by a slight decline, followed by a rebound before continuously decreasing. By contrast, fathers’ PSE followed a linear decrease over time. Finally, our findings evidenced that only the slope of mothers’ PSE negatively predicted adolescents’ RB behaviors at T7, implying that mothers who maintained higher levels of PSE over time had children who later engaged in lower RB behaviors. The study implications are discussed.

## Introduction

The transition from late childhood to late adolescence has long been described as a crucial period in development because it is characterized by major biological, cognitive and psychosocial changes [1, 2]. Along with pubertal development and increasing cognitive maturity, normative changes center around children’s increasing responsibility for their own behaviors and future; moving toward less-dependent and more-mature relationships with family members [2, 3]. In fact, as a way of marking the transition to adulthood, adolescents start to negotiate family rules and degree of supervision and tend to spend more time in settings away from their homes. Due to the increasing independence, the adolescent’s environment grows larger, and numerous opportunities for engaging in rule-breaking (RB) behaviors (e.g., antisocial behaviors, alcohol and drug use) arise [4, 5].

Despite adolescents’ desire for autonomy and increasing distance from the family, parents continue to contribute in central ways to their children’s development and outcomes well beyond the childhood years [6–8]. In fact, researchers describe successful parenting during adolescence as providing a balance between offering adolescents increased autonomy and responsibility while maintaining involvement in their children’s lives [9]. To promote children’s successful transition to adolescence, parents need to feel highly confident in their own ability to influence their adolescents’ behaviors [10, 11]. In this regard, parental self-efficacy has been studied as a pivotal putative mechanism through which parents maintain effective parenting even in the presence of multiple stressors and in the face of aversive and challenging circumstances [12–14].

The concept of Parental Self-Efficacy (PSE) derives from Bandura’s [13, 15] social cognitive theory and refers to convictions about parents’ capacity to cope effectively with the challenges of parenthood and to influence the development of their children positively. PSE is a particular case of perceived self-efficacy which refers to “beliefs in one’s capabilities to organize and execute the courses of action required to produce given levels of attainments” [16, p. 624]. When people think that they can successfully enact a particular behavior, they will also be more likely to display that behavior, especially if they can expect desirable outcomes [13, 17]. Thus, parents who strongly believe in their parenting capabilities are also more likely to be better equipped to handle challenging child behaviors than those who doubt they can influence their child’s development [18–20].

Previous systematic reviews of PSE among parents of children and adolescents have shown that high levels of PSE are associated with adolescents’ successful adjustment and outcomes [see 21, 22–24]. For instance, high levels of PSE have been linked to adolescents’ academic success [12], academic self-regulation [25], and higher competence and well-being [26]. In contrast, low levels of PSE have been related to adolescents’ RB behaviors, such as delinquency and substance use [e.g., 27–30].

Despite the well-conceptualized importance of PSE in predicting child outcomes, most of the available research has relied on cross-sectional data [see 23], and only a few studies have used both mothers’ and fathers’ reports to investigate how PSE relates to child behaviors across time [28, 29, 31]. Moreover, cross-cultural research has showed important differences of fathers’ and mothers’ levels of PSE depending on their cultural background and nationality [32], and research on parental beliefs demonstrates that culture plays an important role in parents’ belief systems [33]. For instance, parents from cultures that place more emphasis on supporting the growth of personal autonomy, such as in the West, allow their adolescent children to make their own decisions, leaving space for PSE to decline as children move into adolescence [e.g., 34]. Similar to most Western cultures, Italian families hold values that reflect the individuation and the development of independence in adolescent children, but also encourage autonomy goals along with strong family ties and interdependence between family members [35, 36]. Hence, as suggested by Manzi et al. [37], the cultural model of Italian families differs from those of other Western cultures in the idea of *autonomy as identity individuation within the family of origin*. In other words, while other Western cultures are more focused on values related to independence starting from early adolescence, such as in the U.S. [38], for Italian adolescents, individuation is represented through freedom of choices that are renegotiated *within* the family with greater egalitarianism as they grow older. Therefore, aspects of connectedness and independence need to be increasingly balanced in Italian parent-child relation [39]. Moreover, in the families of Italian adolescents, mothers still occupy a central role in the elaboration process of educational and occupational aspirations of their children, while fathers maintain a more peripheral position [39]. Hence, the strong interdependence between family members, along with adolescents’ need for independence and mothers’ central role in child development, may differentiate Italian parents from parents of other Western cultures or countries.

Based on the aforementioned considerations, the present study had two major goals: (1) to describe the developmental trends of PSE among Italian mothers and fathers over seven waves (representing children’s transition from late childhood to late adolescence; approximately from 9 to 18 years old); and (2) to examine whether and how initial levels and rates of change of mothers’ and fathers’ PSE predict late adolescents’ RB behaviors (while controlling for the initial levels of RB behaviors at age 9). These goals were also examined while controlling for child gender and family socio-economic status.

### Developmental trends of parental self-efficacy from childhood to adolescence

Parenting tasks vary according to the child’s developmental phases, and as children approach adolescence and start to spend more time outside the home, parents need to hold strong convictions in their ability to exercise their parental role successfully in regard to their children’s educational activities and relations outside the home [13]. In fact, starting from early adolescence, not only are youths exposed to higher academic requirements and goals established by the attendance of middle- and high- school, but also to new social peer contexts that can be a harbinger of potentially risky behaviors [40]. Moreover, with growing independence, adolescents start to establish autonomy and identity and become more inclined to consider family rules as subjective and sometimes arbitrary [7]. During this transitional period, parents cannot exercise direct control and guidance of their children’s behaviors, and may start to feel less efficacious in establishing adequate patterns of supervision crucial for promoting their children’s involvement in educational activities and preventing their engagement in risky behaviors, resulting in declining PSE [41–43].

However, according to Bandura [40], and as recently reviewed by Albanese and colleagues [21], parents who hold high efficacy in keeping track and being involved in their adolescent’s life continue to foster their children’s academic aspirations, help them in school activities, and stay out of trouble much further into childhood, suggesting the importance of parents maintaining high levels of PSE across the child’s transition to adolescence.

Despite the well-known importance of PSE in child development and adjustment, to our knowledge, only three longitudinal studies have investigated the normative developmental trends of PSE, respectively during early [44] and late childhood [45], and early adolescent development [34]. In detail, the three-wave longitudinal study of Weaver and colleagues [44], conducted on a sample of American mother-child dyads, found a trend of increasing PSE from age 2 to 4, evidencing that as experience and familiarity in caring for a young child increase, parents feel increasingly more adept at handling different challenges related to their parenting roles. Similarly, Bi and colleagues [45] conducted a two-and-a-half-year longitudinal study on a sample of Chinese fathers during children’s elementary school-age period and found that fathers’ PSE for educational involvement significantly increased as children moved from the fourth to sixth grade. Differently, Glatz and Buchanan [34] conducted a three-wave longitudinal study on changes in mothers’ and fathers’ PSE among American adolescents from age 11 to 15 and found a significant decrease in PSE over time, suggesting that parents feel gradually less efficacious in their parenting skills as youths gain independence and start to spend more time outside the home. These results were in line with previous cross-sectional studies showing that parents of adolescents report lower levels of PSE than parents of younger children [46, 47].

Although the results of the studies mentioned above illuminate potential differences in PSE based on the child’s age, more research incorporating longer developmental periods and more diverse cultural settings is needed to extend previous findings to under-studied age groups. The challenges of the years leading up to late adolescence highlight the need for parents to face their children’s developmental tasks, such as undergoing critical educational transitions, exploring identity formation, and gaining more independence [48, 49]. Based on the assumption that the advent of these experiences causes changes in the parent-child relationship [50], and that the cultural model of Italian families may differ from those of other Western cultures [38, 51], it becomes crucial to extend the existing literature by investigating whether and how PSE changes in the transition to late adolescence, also in the Italian context.

### Rule-breaking behaviors in the transition to late adolescence

A striking and well-established feature of the adolescence period is increased participation in most forms of RB behaviors that decline as individuals enter adulthood [52, 53]. RB behaviors are defined as covert or non/aggressive behaviors (e.g., vandalism, theft and truancy), and are often considered along with overt or aggressive (AGG) behaviors (e.g., attacking others, fighting and bullying) within the broader construct of antisocial or externalizing behaviors [see 54].

However, previous research has shown that not all adolescents who display the one behavior display the other type, too [53, 55, 56]. First, there is meta-analytic evidence that AGG behaviors are more heritable, and thus more persistent than RB, whereas RB behaviors are more likely to be limited to adolescence and influenced by environmental influences than aggression [57, 58]. In fact, starting from early adolescence, children begin to spend more unsupervised time with their peers, experiencing a social environment that provides increased chances to engage in RB [59]. Second, compared to AGG behaviors, RB behaviors necessitate higher cognitive control and advanced socialization, which often do not emerge until early adolescence [60, 61].

Altogether, these findings suggested that aggressive and RB behaviors show diverse etiologies and contrasting patterns of individual growth across development. Thus, failure to take into account the most appropriate type of externalizing behavior when studying specific child developmental phases could generate misleading conclusions and limit the generalizability of results.

### Parental self-efficacy and adolescents’ rule-breaking behaviors

Parenting is a pivotal contextual factor in fostering or reducing adolescents’ RB behaviors [55]. In fact, parents directly influence child behaviors by the beliefs they hold and the behaviors they exhibit [13].

In regards to PSE, previous research has shown that parents with high levels of parental efficacy are likely to serve as role models for their children, who will adopt their parents’ attitudes and beliefs independently of the parents’ actual behavior [e.g., 12, 62]. However, when it comes to study the associations between PSE and adolescents’ RB behaviors, results differed depending on the study design. While most of previous cross-sectional studies have found support for a direct association between PSE and child behaviors [see 24 for a review], longitudinal research has produced mixed findings, with some showing empirical support for a direct association and some not [28, 29, 31]. For instance, in a longitudinal study examining reciprocal relations between PSE and positive parenting practices in a sample of Mexican American mothers and their children, Dumka and colleagues [28] found that only PSE measured at adolescent age 13 directly predicted less adolescent involvement in externalizing behaviors one year later. Differently, in another study across U.S. adolescents, Glatz and Buchanan [29] found that higher levels of PSE predicted fewer early adolescent externalizing behaviors one year later, but only via promotive parenting practices. Using a sample of Belgian families, Slagt and colleagues [31] found that parents’ PSE assessed at child age 7 did not predict children’s externalizing problems six years later, either directly or indirectly via supportive parenting behaviors and discipline strategies.

The mixed findings between longitudinal studies may be due to many different reasons (i.e., different demographics, ages, study design). For instance, as parents were often reporting on both PSE and parenting practices, it may be possible that parent-reported associations were inflated by shared method variance [63]. More research is needed to understand the direct longitudinal association between PSE and child RB behaviors, especially during longer developmental phases, such as children’s transitions from late childhood to late adolescence.

### Differences in parental self-efficacy among mothers and fathers

In light of historical and societal changes in parenting over the past 50 years [64], children develop in a social context where both mothers and fathers exert influence over their growth and well-being. Hence, many modern fathers, alone and with mothers, play an active and hands-on parenting role in impacting their children’s cognitive and socioemotional development [65, 66].

Although the contemporary view of fathers is that they have important influences on their children’s development through the life course [e.g., 50, 67], the literature on how PSE impacts child developmental outcomes is, for the most part, confined to mothers, and the available studies that have examined mothers’ and fathers’ differences in the associations involving PSE have presented mixed results [see 24]. While some studies have found that mothers have higher levels of PSE, are more involved in specific parenting behaviors and have a greater influence on child outcomes than fathers [27, 29, 68], other studies either have shown similar associations between mothers’ and fathers’ PSE and parenting [69, 70], or between PSE and child outcomes [71, 72]. For instance, de Haan and colleagues [69], in a longitudinal study of Belgium families, showed that parents with higher PSE were also more likely to use positive parenting practices (e.g., warmth and involvement) than parents with a lower sense of efficacy, and that these associations were similar between mothers and fathers. Similarly, results from a two-wave longitudinal study on a sample of Croatian adolescents aged between 11 and 17 years evidenced that, for both mothers and fathers, PSE was linked to antisocial behaviors through less punitive parental practices [72]. The model for fathers also showed a direct effect of PSE on antisocial behaviors, evidencing a partial mediating effect.

Despite the increasing research on the role of fathers in children’s adjustment, the independent contribution of fathers relative to mothers needs additional attention, and more research is needed to understand the associations involving PSE by disentangling maternal from paternal influences.

### Control variables: Child gender and family socio-economic status

Given that the developmental outcomes parents expect and desire for their children and the beliefs parents hold for themselves in achieving those outcomes may vary as a function of the gender of the child [73] and family socio-economic status [i.e., SES; 74], we included child gender and SES as covariates in our model.

Regarding child gender, prior studies reported non-significant differences in PSE as a function of the gender of the child, regardless of whether the sample focused on school-aged or adolescent-aged children [see 23, 24]. Although the majority of these findings came from samples of U.S. parents, and to our knowledge no previous studies have investigated how the gender of the child may affect PSE among Italian parents, there is evidence that Italian mothers and fathers do not hold different perceptions of their parenting role depending on whether the child is a girl or boy [75]. There is also evidence that males engage in higher rates of RB behaviors than females, beginning in childhood and continuing throughout adolescence [76]. As suggested by Moffitt [76], males may be more likely to engage in RB behaviors because they are exposed to greater levels of individual (e.g., novelty-seeking and impulsiveness) and interpersonal (e.g., peer relations) risks than females.

Regarding family SES, as suggested by Bandura [13] and previous findings [12], low-SES families may display higher levels of PSE than high-SES families because PSE is stronger in settings that combine adversities and obstacles. Hence, in the face of scarce resources and demoralizing constraints (e.g., economic hardships), low-SES parents may believe that more efforts are needed to help their children at school and keep them out of troubles, and so believe more in their efficacy as parents compared to wealthy families. Moreover, prior meta-analytic findings [77, 78] evidenced that children from low-SES backgrounds show higher rates of RB behaviors than those from high-SES families. The multiple risk factors associated with lower SES (e.g., economic marginalization, resource scarcity) may contribute to the development of maladaptive behaviors.

### The current study

In the current study, we aimed to extend the existing literature by examining the developmental trends of mothers’ and fathers’ PSE (i.e., parents’ beliefs in their ability to influence their children’s motivations for academic pursuits and help their children to stay out of trouble and avoid risky behaviors), and testing PSE as a predictor of late adolescents’ RB behaviors across seven waves in a sample of 200 Italian mothers, fathers, and their children followed from 9 to 18 years old.

In doing so, we made two predictions:

1. Given the emphasis of Italian families on helping their children developing personal autonomy and individuation while maintaining strong connections with their family of origin as children approach adolescence and start to strive for autonomy [e.g., 37], we hypothesized that the developmental needs of adolescents would gradually push mothers’ and fathers’ PSE to new equilibriums in early and middle-to-late adolescence. Although previous longitudinal data have suggested that PSE decreases as children approach middle adolescence [34], detailed hypotheses about how mothers’ and fathers’ levels of PSE will change from late childhood to late adolescence are hard to formulate due to scarcity of studies conducted on longer developmental periods, especially in the Italian culture. Thus, our examination of how mothers’ and fathers’ levels of PSE will change over time was exploratory.

2. Given the greater influential role of Italian mothers on child behaviors than fathers and mothers of other Western cultures [35], and that RB behaviors are associated with low levels of PSE [27, 52], we hypothesized that, even during a challenging time characterized by greater child independence and autonomy, mothers who maintain higher levels of PSE over time will have children who engage in lower RB behaviors during late adolescence, while controlling for the initial levels of child RB behaviors. However, given that recent research has evidenced similar associations between mothers’ and fathers’ PSE and child outcomes [72], we expected to find this association also for fathers, although with less strong effects.

Finally, regarding gender, as previous studies evidenced that child gender does not have a statistically significant influence on PSE (for an overview, see [23]), whereas RB behaviors have been found to be more common in males than females [76], we expected that levels of mothers’ and fathers’ PSE would not differ based on the gender of the child, whereas males will be higher in their levels of RB behaviors than females.

Regarding SES, as suggest by existing literature [e.g., 12, 78], we expected to find a negative association between SES and mothers’ and fathers’ PSE, such that low-SES families will display higher levels of PSE than high-SES families, as well as between SES and RB behaviors, such that youths coming from low-SES families will display higher levels of RB behaviors.

## Materials and methods

### Participants

Participants included 200 Italian children (*M*_AgeAtTime1_ = 9.80, *SD* = 0.65; 50.5% girls), their mothers (*N* = 200, *M*_AgeAtTime1_ = 40.31, *SD* = 5.33), and their fathers (*N* = 190, *M*_AgeAtTime1_ = 43.13, *SD* = 6.01) in the larger longitudinal and cross-cultural study Parenting Across Cultures (PAC; e.g., [79]). As reported in Table 1, data for this study were collected in seven waves between 2010 and 2018, when child participants were approximately aged 9–18; Time 1 (T1–2010) corresponds approximately to child age 9, Time 2 (T2–2011) to age 10, Time 3 (T3–2014) to age 13, Time 4 (T4–2015) to age 14, Time 5 (T5–2016) to age 15, Time 6 (T6–2017) to age 16, and Time 7 (T7–2018) to age 17–18. There was about a one-year interval between each time-point, except between T2 and T3, in which there was a three-year interval (data from this interval were missing by design). Data were accessed for research purposes from November 2019 to January 2023.

**Table 1:** Means and standard deviations of the age of mothers, fathers, and children across the seven waves.

	T1–2010	T2–2011		T3–2014	T4–2015	T5–2016	T6–2017	T7–2018
	Age, mean (*SD*)	Age, mean (*SD*)		Age, mean (*SD*)	Age, mean (*SD*)	Age, mean (*SD*)	Age, mean (*SD*)	Age, mean (*SD*)
Mothers	40.31 (5.33)	41.53 (5.28)	Missing by design	44.01 (5.43)	45.35 (5.30)	46.35 (5.30)	47.50 (5.59)	48.85 (5.48)
Fathers	43.13 (6.01)	44.34 (6.34)		46.98 (5.86)	48.15 (6.06)	49.15 (6.06)	50.92 (5.57)	52.65 (5.76)
Child	9.80 (0.65)	10.93 (0.61)		13.54 (0.62)	14.65 (0.63)	15.58 (0.63)	16.68 (0.68)	18.33 (0.64)

*Note*: *SD* = Standard Deviation. Mothers’ and fathers’ reports of their parental self-efficacy were assessed from T1 to T7, and child reports of their rule-breaking behaviors were assessed only at T1 and T7.

Participants were recruited from two sites in Italy, Naples and Rome (details about sample size across measurement time points in the two sites is reported in S1 Table). At T1, most parents were married or cohabiting (86.9%) and had approximately a high school education (Mothers; *M*_*EducationYears*_ = 11.94, *SD* = 4.46; Fathers; *M*_*EducationYears*_ = 12.14, *SD* = 4.31). At T1, 60.6% of mothers were employed, whereas 94.4% of fathers were employed. Only 35.3% of the sample reported having a low gross annual household income (less than €16,000).

Participants’ participation rate remained high across time. Specifically, from T1 to T7, mothers’ participation rate was 87.5%, fathers’ was 86.1%, and adolescents’ was 89.4%. The attrition rate was principally due to two main reasons: the unavailability of the families to participate in the later data collection or their refusal to participate in that specific wave.

### Procedure

Letters describing the study were sent home to families, and parents were asked to return a signed form if they agreed to be contacted further. After obtaining parental written informed consent, and child assent, questionnaires were completed in the participants’ home or locations of their choosing (e.g., school, home). Mothers, fathers, and children completed interviews either orally or as written questionnaires. Testing sessions lasted approximately two hours. Families received modest financial compensation for their participation. The Declaration of Helsinki was adequately addressed, and the study received written approval from the Institutional Review Board (IRB) of the Department of Psychology at Sapienza University of Rome.

### Measures

#### Demographic variables

Child gender (0  =  boys, 1  =  girls) and family socio-economic status (SES) at T1 were used as covariates. According to Bollen and Lennox [80], SES was treated as a formative factor composed of three indicators: mothers’ educational levels, father’s educational levels, and family income. Education was measured as years of parental education. Family income was measured on 1–10 scale (1 = up to €5,000; 2 = between €5,000 and €10,000; 3 = between €11,000 and €15,000; 4 = between €16,000 and €29,000; 5 = between €30,000 and €40,000; 6 = between €41,000 and €50,000; 7 = between €51,000 and €60,000; 8 = between €61,000 and €70,000; 9 = between €71,000 and €80,000; and 10 = beyond €81,000).

#### Parental self-efficacy (from T1 to T7; mother and father report)

Parents’ beliefs of their parenting capabilities were assessed using the 6-item Parental Self-Efficacy Scale (PSE; [81–83]. The PSE scale measures two dimensions of self-efficacy, parents’ efficacy beliefs regarding influence over their children’s motivations for academic pursuits (three items, e.g., “How much can you do to make your children see school as valuable?”) and parents’ efficacy beliefs regarding their ability to help their children to stay out of trouble and avoid risky behaviors (three items, e.g.,“How much can you do to keep track of what your children are doing when they are outside the home?”). Parents rated the strength of their self-efficacy beliefs on a 5-point Likert scale (ranging from 1 = *nothing* to 5 = *a great deal)*. Mother- and father-rated scales were each computed as the average of 6 items, with higher scores indicating higher levels of PSE. The psychometric properties of the scale have been assessed on two samples of Italian respondents [83]. This Italian study highlighted that the instrument tapped into a single dimension of parental self-efficacy and that it had high construct validity. McDonald’s Omegas coefficients for T1, T2, T3, T4, T5, T6, and T7 were 0.71, 0.78, 0.75, 0.81, 0.72, 0.80, and 0.79 for mothers, and 0.83, 0.85, 0.84, 0.90, 0.87, 0.87, and 0.88 for fathers. Analyses demonstrated measurement invariance for this scale across mothers and fathers (see S2 and S3 Tables for details).

#### Adolescents’ rule-breaking behaviors (T1 and T7; child report)

Adolescents’ rule‐breaking behaviors were measured utilizing the 11-item rule‐breaking behavior subscale (RB) of the Youth (T1) and Adult (T7) Self-Report (YSR, [84]; ASR, [85]). At both T1 and T7, youths were asked to rate how true each item was during the last six months on a three‐point Likert scale (0 = *not true*, 1 = *somewhat or sometimes true*, 2 = *very or often true*).

Child-rated scale was computed as the average of 11 items, with higher scores indicating higher RB behaviors. Examples of items are: “I hang out with kids who get in trouble,” “I steal things at home,” and “I lie or cheat.” The Achenbach measures are among the most widely used instruments in international research, with translations in over 100 languages and strong, well-documented psychometric properties (e.g., [86]). McDonald’s Omegas coefficients in this study were 0.82 at T1 and 0.68 at T7.

### Data analytic approach

At a preliminary level, we computed descriptive statistics (means, standard deviations, skewness, and kurtosis), Pearson’s *r* correlations among all study variables, within and across the seven-time points, and independent-samples t-tests to verify child gender effects on the scores of all PSE and RB variables. We also modeled SES as a formative latent factor composed of three observed indicators: mothers’ educational levels, father’s educational levels, and family income. A formative latent variable is created by using a weighted sum of several indicators that cause or form the construct (i.e., the indicators cause the latent construct; [80, 87]).

Subsequently, we followed a three-step approach to examine the longitudinal changes of mothers’ and fathers’ PSE and its impact on late adolescents’ RB behaviors. First, we tested the longitudinal measurement invariance of the six-item PSE scale both for mothers and fathers. Because an important prerequisite for modeling mean-level changes is to ensure that the instruments are consistent in measuring the same construct of interest over time (e.g., [88]), we aimed to reach at least strong longitudinal measurement invariance (i.e., scalar invariance) for the PSE scale. We preliminarily estimated a configural invariance model in which the same pattern of free-factor loadings was specified across time. Next, we tested the metric (or weak) invariance of the PSE scale by constraining the unstandardized factor loadings of each item to be equal over time. Finally, to test scalar invariance, the items were fixed to have the same origins (i.e., the intercepts) over time. To identify our latent variables, we fixed the intercept of the factor to 0. Modification indices were used to identify potential noninvariant items and refine the models to reach at least partial measurement equivalence [88]. Measurement invariance across time was examined by using the changes in CFI (ΔCFI) with a critical level of 0.01 [89].

Second, to test our first hypothesis, we performed a series of unconditional Latent Growth Curve Models (LGCMs; [90]), in which we estimated two latent growth parameters: (1) the intercept, which represented the initial level of PSE (T1) and (2) the slope, which represented the rate of change over time (from T1 to T7). These model were performed within the Structural Equation Modeling framework [91] and were tested separately for mothers and fathers.

To identify the best fitting trend, we used the Δχ^2^ of six (nested) unconditional models: (1) a random-intercept only no growth model, (2) a linear growth model which included the intercept and linear slopes, (3) a quadratic growth model with two latent factors of change estimates, namely the linear and quadratic trends, (4) a cubic growth model, with three latent factors of change estimates, namely the linear, quadratic and cubic trends, (5) two piecewise linear growth models, with one intercept and two linear slopes each, and (6) a freed factor loading growth model, with one intercept factor and a freely estimated slope factor. For models 1, 2, 3 and 4, factor loadings for the intercept were fixed at 1 at all seven time points, and factor loadings for the slope were fixed at 0, 1, 4, 5, 6, 7, and 8 (representing one-year spaced assessment between all-time points, except for three years spaced assessments between T2 and T3). Factor loadings for the slope were reparametrized for model 3 and 4 to include quadratic and cubic terms, respectively. Model 4 was estimated to allow trajectory shapes to change across time, and quadratic and cubic variances were fixed to zero within groups to allow for model estimation [92]. For model 5, given that at least three time points are needed to identify each piece (see [90, 93]), and that we did not have a-priori hypotheses of where to place the “knot” or “turning point”, two piecewise models were developed: model 5a with a knot placed at T3 (i.e., approximately at child age 13) and model 5b with a knot placed at T4 (i.e., approximately at child age 14). When the knot was placed at T3, the loadings for the first slope factor were set to 0 (T1), 1 (T2), 4 (T3), 4 (T4), 4 (T5), 4 (T6), 4 (T7) and the loadings for the second slope factor were set to 0 (T1), 0 (T2), 0 (T3), 1(T4), 2 (T5), 3 (T6), and 4 (T7). When the knot was placed at T4, the loadings for the first slope factor were set to 0 (T1), 1 (T2), 4 (T3), 5 (T4), 5 (T5), 5 (T6), 5 (T7) and the loadings for the second slope factor were set to 0 (T1), 0 (T2), 0 (T3), 0 (T4), 1 (T5), 2 (T6), and 3 (T7). Finally, for model 6, to freely capture the total change that occurred over all time points, we fixed the first loading of the slope factor to 0 and the last to 1.

To compare models’ fit, we used the corrected Satorra-Bentler chi-square difference test (Δχ^2^) for the nested model [94].

Third, once we identified the best unconditional LGCM, and in line with our second hypothesis, we tested two conditional LGCMs (one for mothers and one for fathers) in which we considered adolescents’ RB behaviors at T7 as the outcome variable, while controlling for the initial levels of RB behaviors at T1, child gender, and family SES. We estimated all parameters and handled missing data using the Maximum Likelihood with standard errors robust to non-normality (MLR) estimator in Mplus 8.4 [95]. To evaluate the goodness of fit of our models, we used the following indicators: Chi-square index (χ^2^), Comparative-Fit-Index (CFI), and Tucker-Lewis-Index values greater than 0.90 as indicators of acceptable model fit, and CFI/TLI >0.95 as indicators of good model fit [96], as well as Root-Mean-Square-Error-of-Approximation (RMSEA) with 90% Confidence Interval (CI), and Standardized-Root-Mean-Square-Residual (SRMR) values lower than 0.08 as indicators of moderate model fit and below 0.05 as an indicator of good model fit [96, 97].

## Results

### Preliminary analyses

Means, standard deviations and Pearson’s *r* correlations among the observed mean scores were reported for each PSE and RB scale variable over time (see Table 2). Standardized factor loadings for the SES formative latent factor were 0.450, 0.440, and 0.225 for mothers’ levels of education, fathers’ levels of education, and family income, respectively, and were all significant (*p* < 0.001). As shown in Table 2, most of the correlations between mothers’ PSE and adolescents’ RB behaviors at T7 were negative and statistically significant. Fathers’ PSE at T3 and later was significantly associated with T7 RB behaviors. In addition, the positive correlations among PSE from T1 to T7 attested to a moderate degree of rank-order stability over time, for both mothers’ and fathers’ beliefs. Results of independent-samples t-tests showed that significant gender differences emerged only in RB behaviors at T1, *t*(178.337) = 2.858, *p* < 0.05, *d* = 0.419, and this effect was small (see [98]). Males (*M* = 0.207, *SD* = 0.158) obtained higher scores than females (*M* = 0.145, *SD* = 0.138). Mothers’ and fathers’ years of education and family income were negatively associated with mothers’ and fathers’ PSE over time and child RB behaviors at T1, whereas mothers’ years of education were positively associated with child RB behaviors at T7. Independent samples t-tests were run to test differences between boys’ and girls’ RB behaviors.

**Table 2:** Descriptive statistics (mean and SD) and correlations (coefficient r Pearson) for all study variables.

*Variables*	Mean (SD)	(1)	(2)	(3)	(4)	(5)	(6)	(7)	(8)	(9)	(10)	(11)	(12)	(13)	(14)	(15)	(16)	(17)	(18)	(20)
(1) Child Gender	– (–)	-																		
(2) M_Education	– (–)	-0.029	-																	
(3) F_Education	– (–)	0.040	0.733**	-																
(4) SES	– (–)	-0.073	0.649**	0.641**	-															
(5) PSE-M T1	3.80 (0.51)	-0.034	-0.290**	-0.208**	-0.185*	-														
(6) PSE-M T2	3.73 (0.55)	0.053	-0.264**	-0.278**	-0.177*	0.549**	-				-									
(7) PSE-M T3	3.61 (0.54)	0.015	-0.271**	-0.200**	-0.193**	0.457**	0.603**	-												
(8) PSE-M T4	3.67 (0.58)	0.074	-0.375**	-0.288**	-0.255**	0.441**	0.492**	0.582**	-											
(9) PSE-M T5	3.62 (0.53)	0.041	-0.306**	-0.227**	-0.197**	0.444**	0.533**	0.613**	0.664**	-										
(10) PSE-M T6	3.71 (0.58)	0.054	-0.265**	-0.213**	-0.268**	0.382**	0.487**	0.608**	0.634**	0.639**	-									
(11) PSE-M T7	3.55 (0.61)	0.077	-0.222**	-0.195*	-0.164*	0.269**	0.424**	0.490**	0.486**	0.561**	0.572**	-								
(12) PSE-F T1	3.71 (0.62)	0.046	-0.264**	-0.249**	-0.279**	0.192*	0.269**	0.207*	0.350**	0.169*	0.213*	0.268**	-							
(13) PSE-F T2	3.69 (0.63)	-0.002	-0.256**	-0.251**	-0.218**	0.185*	0.140	0.223**	0.217**	0.152	0.178*	0.158	0.661**	-						
(14) PSE-F T3	3.60 (0.63)	0.009	-0.283**	-0.311**	-0.191*	0.272**	0.290**	0.330**	0.395**	0.297**	0.279**	0.284**	0.666**	0.608**	-					
(15) PSE-F T4	3.61 (0.60)	-0.049	-0.300**	-0.294**	-0.163*	0.218**	0.235**	0.238**	0.291**	0.244**	0.237**	0.292**	0.703**	0.648**	0.723**	-				
(16) PSE-F T5	3.49 (0.65)	-0.05	-0.263**	-0.272**	-0.228**	0.141	0.239**	0.311**	0.283**	0.229**	0.254**	0.254**	0.675**	0.658**	0.735**	0.816**	-			
(17) PSE-F T6	3.52 (0.63)	-0.037	-0.302**	-0.249**	-0.283**	0.106	0.214*	0.273**	0.298**	0.142	0.224**	0.180*	0.689**	0.628**	0.701**	0.670**	0.757**	-		
(18) PSE-F T7	3.47 (0.67)	0.081	-0.240**	-0.173*	-0.147	0.113	0.228**	0.218*	0.312**	0.173*	0.285**	0.283**	0.536**	0.494**	0.617**	0.571**	0.643**	0.692**	-	
(19) RB-C T1	0.18 (0.16)	-0.207**	-0.172*	-0.195**	-0.120	0.034	0.001	-0.106	-0.008	-0.089	-0.074	-0.035	-0.028	-0.022	0.006	0.008	-0.076	-0.147	-0.117	-
(20) RB-C T7	0.36 (0.25)	-0.002	0.201**	0.152	0.166*	-0.114	-0.023	-0.205**	-0.201**	-0.253**	-0.203**	-0.242**	-0.124	-0.095	-0.208*	-0.234**	-0.184*	-0.231**	-0.264**	0.168*

*Note*. Child gender was coded 0 = boys, 1 = girls. M_Education = years of mothers’ education, F_Education = years of fathers’ education. SES was treated as a formative latent factor composed of mothers’ and father’s levels of education and family income. PSE-M = Mothers’ parental self-efficacy; PSE-F = Fathers’ parental self-efficacy; RB-C = Child rule-breaking behaviors. *SD* = Standard deviation.

** = *p* < .01

* = *p* < .05.

## Unconditional latent growth curve models

To identify the best fitting developmental trends of mothers’ and fathers’ PSE from late childhood to late adolescence, we tested six (nested) unconditional LGCM for each parent: no growth, linear, quadratic, and cubic change (see the Data Analytic Approach section for further details). Fit indices and the comparison between each model are summarized in Table 3.

**Table 3:** Model fit indices of the univariate latent growth curve models (LGCMs) for mothers’ and fathers’ parental self-efficacy (PSE).

	χ^2^	*df*	*scf*	*p*	CFI	TLI	RMSEA (90%CI)	SRMR	MC	Δχ^2^	Δ*df*	*p*
**Mothers**												
1. No Growth	88.576	26	1.042	<0.001	0.871	0.896	0.108 (0.083 0.133)	0.261				
2. Linear	37.134	23	1.084	<0.05	0.971	0.973	0.054 (0.016 0.085)	0.115	2 vs 1	72.282	3	<0.001
3. Quadratic	29.095	19	1.088	0.065	0.979	0.977	0.051 (0.000 0.085)	0.059	3 vs 2	8.073	4	0.089
**4. Cubic**	**23.026**	**18**	**1.087**	**0.189**	**0.990**	**0.988**	**0.037 (0.000 0.076)**	**0.046**	**4 vs 2**	**14.186**	5	**<0.05**
5. Piecewise	30.203	19	1.064	0.049	0.977	0.975	0.053 (0.003 0.088)	0.069	5a vs 2	6.885	4	0.142
	31.270	19	1.073	0.037	0.975	0.972	0.056 (0.013 0.090)	0.072	5b vs 2	5.897	4	0.207
6. Freed Factor Loading	26.386	18	1.057	0.091	0.983	0.980	0.047 (0.000 0.084)	0.070	6 vs 2	10.467	5	0.063
**Fathers**												
1. No Growth	85.140	26	1.011	< .001	0.900	0.919	0.113 (0.087 .0140)	0.140				
**2. Linear**	**29.257**	**23**	**0.992**	**0.172**	**0.989**	**0.990**	**0.039 (0.000 0.077)**	**0.076**	**5 vs 4**	**49.326**	**3**	**<0.001**
3. Quadratic	22.793	19	0.984	0.247	0.994	0.993	0.033 (0.000 0.077)	0.080	6 vs 5	6.403	4	0.171
4. Cubic	22.779	18	0.983	0.199	0.992	0.991	0.039 (0.000 0.081)	0.079	7 vs 5	6.473	5	0.263
5. Piecewise	22.636	19	0.977	0.254	0.994	0.993	0.033 (0.000 0.076)	0.079	5a vs 2	6.497	4	0.165
	24.789	19	0.984	0.168	0.990	0.989	0.041 (0.000 0.082)	0.076	5b vs 2	4.496	4	0.343
6. Freed Factor Loading	28.312	18	0.910	0.057	0.983	0.980	0.057 (0.000 0.095)	0.069	6 vs 2	2.532	5	0.772

*Note*. The best fitting developmental trends of mothers’ and fathers’ PSE are reported in bold. Regarding piecewise models, for both mothers and fathers, the first model (5a) refers to the model in which the knot was place at T3 (child age 13), whereas the second one (5b) refers to the model in which the knot was place at T4 (child age 14). The following fit indexes are reported: χ^2^ = chi square; *df* = degrees of freedom; *scf* = scaling correction factor; *p* = χ2 p-value; CFI = Comparative-fit-index; TLI = Tucker-Lewis-index; RMSEA = Root-mean-square-error-of-approximation with 90% confidence intervals (90% CI); SRMR = Standardized-Root-Mean-Square-Residual; MC = model comparison; Δχ2 = chi square difference test; Δdf = degrees of freedom difference; *p* = Δχ2 p-value

Regarding mothers’ PSE, the cubic model was the model that best fitted our data, indicating an overall trend of decline-increase-decline of mothers’ PSE over time. The significant mean of the intercept (*M*_*i*_) (*M*_*i*_ = 3.810, *p* < 0.001) showed that mothers reported a positive average starting point different from zero at T1, and the significant variance of the intercept (*s*_*i*_^*2*^) (*s*_*i*_^*2*^ = 0.148, *p* < 0.001) indicated inter-individual variability around this mean. The mean of the linear slope (*M*_*ls*_) was significant and negative (*M*_*ls*_ = -0.140, *p* < 0.001), indicating that mothers’ PSE tended to decline in a linear fashion over time. The non-significant variance of the linear slope (*s*_*ls*_^*2*^) (*s*_*ls*_^*2*^ = 0.007, *p* = 0.245) indicated no inter-individual variability in change over time. The covariance between the intercept and the linear slope (I-S_l_-Cov) was non-significant (I-S_l_-Cov = 0.001, *p* = 0.932), indicating that initial levels of mothers’ PSE were not associated with their linear decrease over time. The mean of the quadratic slope (*M*_*qs*_) was significant and positive (*M*_*qs*_ = 0.033, *p* < 0.01), suggesting a pattern reflecting a U-shaped growth trend of mothers’ PSE. The quadratic trend was not statistically different from the linear model (see Table 3). The quadratic trend did not add valuable information regarding the change of mothers’ PSE, with a non-significant variance of the quadratic trend (*s*_*qs*_^*2*^) (*s*_*qs*_^*2*^ = 0.000, *p* = 0.526) and a non-significant covariance with the intercept (I-Q-Cov = -0.001, *p* = 0.418) and with the quadratic slope (S-Q-Cov = -0.001, *p* = 0.465). A cubic growth change was captured from T1 to T7 as indicated by the significant and negative mean of the cubic slope (*M*_*cs*_) (*M*_*cs*_ = -0.002, *p* < 0.05), suggesting an overall trend characterized initially by a slight decline (from late childhood to early adolescence; approximately from 9 to 13 years old), followed by a rebound (during middle adolescence; approximately from 14 to 15 years old) before continuously decreasing (as children moved into late adolescence and emerging adulthood; approximately from 16 to 18 years old). For identification purposes, the variance of the cubic slope was fixed to zero. The cubic trend was statistically different from the linear model. The two piecewise and the freed factor loading models did not add valuable information regarding the change of mothers’ PSE over time.

Regarding fathers’ PSE, linear, quadratic, and cubic models showed acceptable fit indices; however, the linear model was chosen over the no-growth, the quadratic, and the cubic model because it was the model that best represented our data. The significant mean of the intercept (*M*_*i*_ = 3.719, *p* < 0.001) showed that fathers reported a positive average starting point different from zero at T1, and the significant variance of the intercept (*s*_*i*_^*2*^ = 0.278, *p* < 0.001) indicated inter-individual variability around this mean. Moreover, the mean of the linear slope was significant and negative (*M*_*ls*_ = -0.031, *p* < 0.001), indicating that fathers’ PSE showed a decrease from T1 to T7, with inter-individual variability in growth over time, as shown by the significant variance of the slope (*s*_*ls*_^*2*^ = 0.002, *p* < 0.01). Finally, the covariance between the intercept and the slope was non-significant (I-S_l_-Cov = -0.004, *p* = 0.229), indicating that initial levels of fathers’ PSE were not associated with their linear decrease over time. The two piecewise and the freed factor loading models did not add valuable information regarding the change of fathers’ PSE over time.

**Fig 1:**
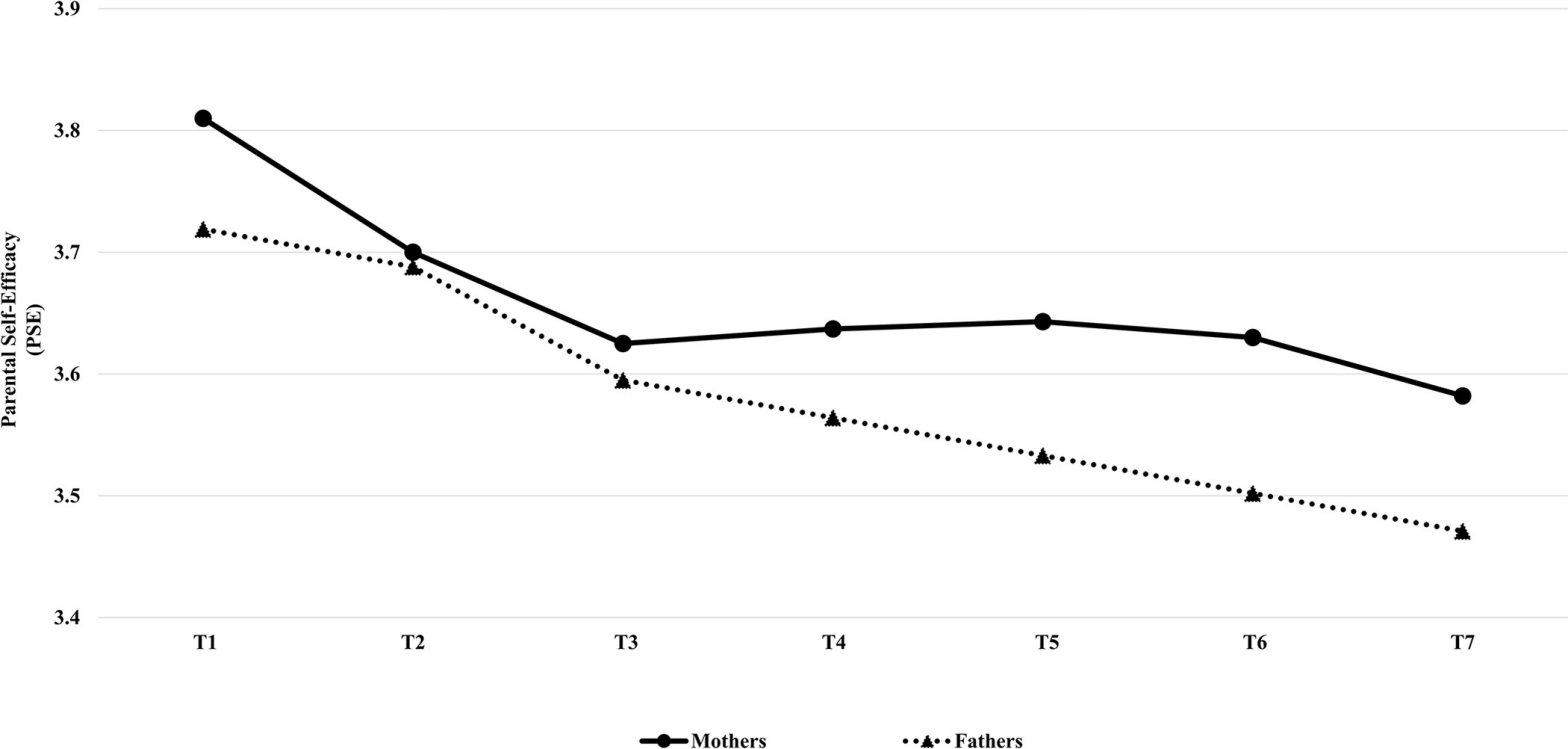
Developmental trends of mothers’ and fathers’ parental self-efficacy (PSE) from T1 to T7. Time 1 (T1) corresponds approximately to child age 9, Time 2 (T2) to age 10, Time 3 (T3) to age 13, Time 4 (T4) to age 14, Time 5 (T5) to age 15, Time 6 (T6) to age 16, and Time 7 (T7) to age 17–18.

The developmental trends of mothers’ and fathers’ PSE are depicted in Fig 1 (see S1 and S2 Figs for factor loadings’ information regarding the best-fitting univariate LGCMs for mothers and fathers, respectively).

### Conditional latent growth curve models

**Fig 2:**
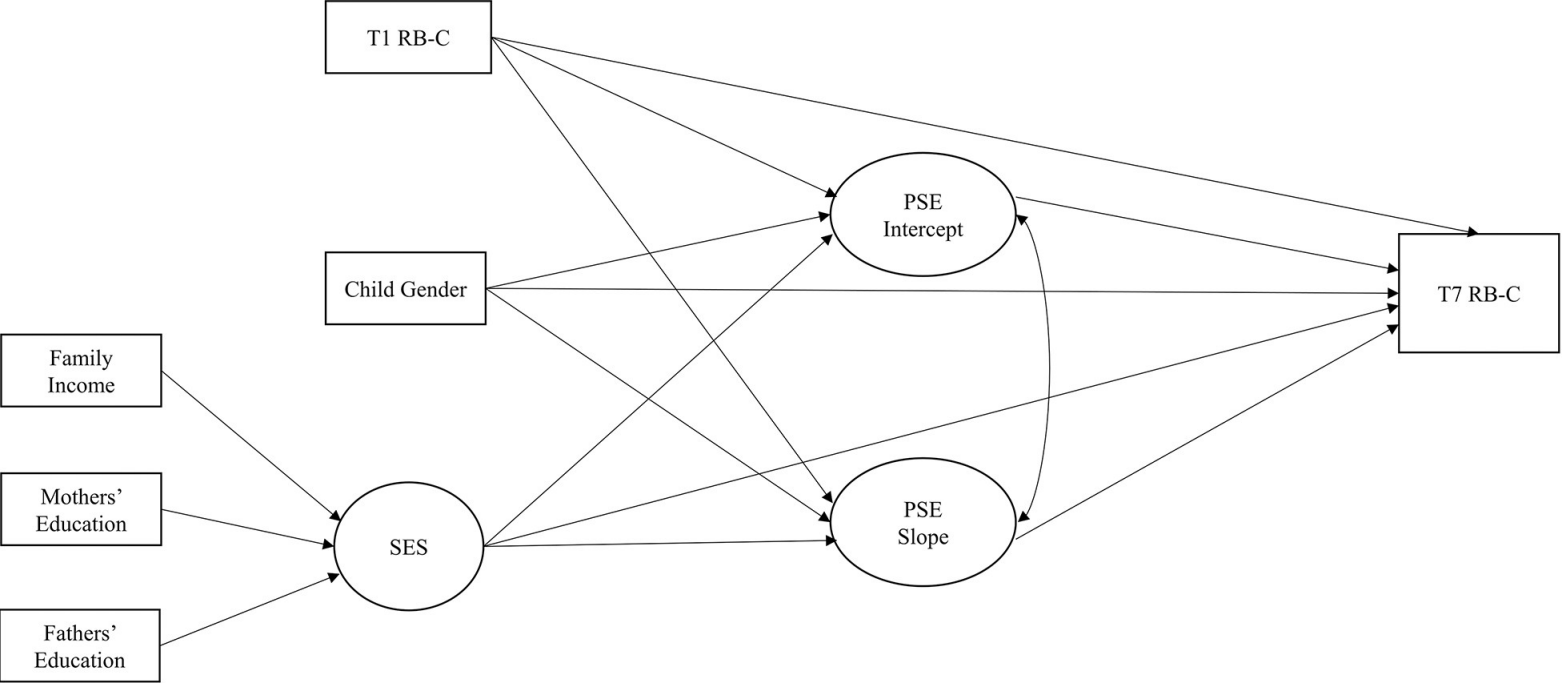
Hypothesized Conditional latent growth curve models (LGCMs). This model was implemented separately for mothers and fathers. We have used the cubic slope for mothers’ model and the linear slope for fathers’ model. Rectangles are measured variables, and circles are latent variables. T1 = Time 1; T7 = Time 7. Child gender was coded 0 = boys, 1 = girls. SES was treated as a formative latent factor composed of mothers’ and fathers’ levels of education and family income. PSE = Parental self-efficacy; RB-C = Child Rule-Breaking Behaviors.

To evaluate whether and how initial levels (i.e., intercepts) and rates of change (i.e., slopes) of mothers’ and fathers’ PSE were associated with adolescents’ RB behaviors at T7, we tested two conditional LGCMs (one for mothers and one for fathers) while controlling for the initial levels of RB behaviors (at T1), child gender (0 = boys, 1 = girls), and SES (treated as a formative factor composed of three indicators: mothers’ educational levels, fathers’ educational levels, and family income; see Fig 2).

Among mothers, the model showed a good fit to the data [χ^2^ (57) = 49.930, *p* = 0.735, CFI = 1.000, TLI = 1.000, RMSEA = 0.000 (0.000 0.035); SRMR = 0.070]. As shown in Table 4, we found a significant and negative effect of the slope of mothers’ PSE on RB behaviors at T7 while controlling for the stability of RB behaviors at T1. This finding indicated that less of a decline in mothers’ PSE over time (i.e., cubic trend) predicted lower RB behaviors at T7, thereby highlighting the protective role of maternal self-efficacy in preventing late adolescents’ engagement in RB behaviors. Moreover, SES negatively predicted the intercept and positively predicted RB behaviors at T7, meaning that families with higher SES reported lower initial levels of PSE and that adolescents from high-SES families were more likely to engage in RB behaviors than adolescents from low-SES families. Finally, the covariation between PSE slope and intercept was negative and significant, indicating that mothers who exhibited higher levels of PSE at T1 decreased less in their parental efficacy over time. Overall, the model accounted for 20% of the variance of adolescents’ RB behaviors at T7.

**Table 4:** Conditional latent growth curve models (CLGCMs).

* *	Mothers			Fathers		
*Predictors of T7 RB-C Behaviors*	*SE*	*β*	CI (95%)	*SE*	*β*	CI (95%)
PSE Intercept → T7 RB-C	0.081	-0.059	[-0.218, 0.101]	0.086	-0.126	[-0.29, 0.05]
PSE Slope → T7 RB-C	0.098	-0.331*	[-0.523, -0.139]	0.120	-0.145	[-0.38, 0.09]
T2 RB-C → T7 RB-C	0.081	0.206*	[0.048, 0.365]	0.081	0.212*	[0.06, 0.37]
Child Gender → T7 RB-C	0.080	-0.018	[-0.175, 0.140]	0.083	-0.034	[-0.19, 0.13]
SES → T7 RB-C	0.092	0.221*	[0.041, 0.401]	0.090	0.203*	[0.04, 0.39]
*Predictors of Intercept and Slope*						
T1 RB-C → PSE Intercept	0.092	-0.074	[-0.254, 0.106]	0.095	-0.077	[-0.263, 0.109]
T1 RB-C → PSE Slope	0.129	-0.069	[-0.321, 0.183]	0.201	-0.086	[-0.480, 0.308]
Child Gender → PSE Intercept	0.086	-0.006	[-0.174, 0.163]	0.083	-0.037	[-0.200, 0.125]
Child Gender → PSE Slope	0.119	0.067	[-0.167, 0.301]	0.139	0.063	[-0.209, 0.334]
SES → PSE Intercept	0.073	-0.370**	[-0.513, -0.228]	0.074	-0.352***	[-0.498, -0.206]
SES → PSE Slope	0.103	-0.028	[-0.230, 0.175]	0.136	-0.023	[-0.290, -0.244]
*Correlations*						
PSE Intercept ↔ PSE Slope	0.136	-0.337*	[-0.602, -0.071]	0.153	-0.264	[-0.564, 0.036]

*Note*. We have used the cubic slope for mothers’ model and the linear slope for fathers’ model. Standard Error (*SE*), Standardized betas (*β)* (→),95% confidence intervals (CI) and correlation coefficients (↔) are reported. Child gender was coded 0 = boys, 1 = girls. SES was treated as a formative latent factor composed of mothers’ and fathers’ levels of education and family income. PSE-M = Mothers’ parental self-efficacy; PSE-F = Fathers’ parental self-efficacy; RB-C = Child rule-breaking behaviors.

** = *p* < .01

* = *p* < .05.

Among fathers, the model showed a good fit to the data [χ^2^ (59) = 64.036, *p* = 0.3043, CFI = 0.992, TLI = 0.991, RMSEA = 0.022 (0.000 0.053); SRMR = 0.057]. As shown in Table 4, neither the intercept nor the slope of fathers’ PSE significantly predicted RB behaviors at T7. However, as for mothers, SES negatively predicted the intercept and positively predicted RB behaviors at T7. Finally, the covariation between the slope and intercept was negative, but nonsignificant. Overall, the model accounted for 13% of the variance of adolescents’ RB behaviors at T7.

## Discussion

The purpose of this longitudinal study was two-fold. First, we examined the developmental trends of parental self-efficacy (PSE) among Italian mothers and fathers over seven waves (representing children’s transition from late childhood to late adolescence; approximately from 9 to 18 years old). Second, we examined whether and how initial levels and rates of change of mothers’ and fathers’ PSE predicted late adolescents’ rule-breaking (RB) behaviors. These aims were also examined while controlling for the initial levels of RB behaviors, child gender, and family SES. Overall, the results of this study add and move beyond the understanding of previous short-term longitudinal studies on the development of PSE and its protective function as children progress into late adolescence.

Overall, findings of this study showed that mothers’ and fathers’ PSE followed different developmental trends over time. Mothers’ PSE showed a cubic trend of decline-increase-decline, whereas fathers’ PSE followed a linear decreasing trend. Moreover, mothers showing steeper decreases in PSE over time had children with higher RB behaviors at age 18, implying that mothers who maintained higher levels of PSE over time had children who engaged in lower RB behaviors, and as mothers’ PSE declined their children’s RB behaviors at age 18 increased. However, these results were not replicated for fathers.

### Developmental trends of mothers’ and fathers’ PSE

With regard to the primary goal of this study, the average developmental course of PSE among mothers and fathers was one of decline. These results were in line with previous findings showing a trend of decline in PSE as a function of child age [34] and may suggest that as children move toward adolescence and gradually strive for more independence, thereby taking more distance from their parents, mothers and fathers may question their efficacy and lower their expectations of being able to influence the development of their children positively. In fact, consistent with the literature [99, 100], starting in early adolescence, the primary bond of the child with mother and father changes into a more equal relationship, and the increasing need for autonomy may leave more room for mothers’ and fathers’ PSE to decline as youths become young adults.

However, although the decline in fathers’ PSE was persistent from late childhood through late adolescence, mothers’ PSE did not decline linearly across adolescence, but changed curvilinearly. Mothers’ PSE followed a cubic trend that revealed a decreasing pattern characterized initially by a slight decline (from late childhood to early adolescence; approximately from 9 to 13 years old), followed by a rebound (during middle adolescence; approximately from 14 to 15 years old) before continuously decreasing (as children moved into late adolescence and emerging adulthood; approximately from 16 to 18 years old). A possible explanation for the different developmental trends of mothers’ and fathers’ PSE may derive from cultural-based values and beliefs shared by parents in Italian families. As mentioned earlier, Italian mothers are more prone to recognize, give importance to, and deal with their children’s requests, and educational and relational needs compared to fathers [51, 101]. Thus, certain transitions in the development of the child (e.g., puberty, finishing school) may be perceived as more challenging by mothers than fathers, thus causing temporary perturbations–as evinced by the rebound of the slope—in mothers’ sense of efficacy. As middle adolescence corresponds to Italian youths’ passage from middle to high school (approximately at child age 14) and coincides with a period when most adolescents experience significant changes at both the biological, individual and social environmental levels [62], mothers may feel that more effort is needed to help their children facing their challenging developmental phases, resulting in a boost of their sense of efficacy that declines as they learn to adapt their parenting beliefs and practices to the increasing maturity and changing needs of the growing child. However, the explanation for mothers’ PSE trend is speculative and more longitudinal research with the inclusion of time-varying covariates (e.g., child pubertal status) is necessary to understand the growth rate variation of mothers’ PSE, especially during the middle adolescence period.

### PSE and adolescents’ RB behaviors

With regard to the second goal of this study and in partial accordance with our second hypothesis, we found that a smaller decline in mothers’ PSE predicted fewer adolescent RB behaviors at age 18 (T7), even while controlling for the strong stability of RB behaviors at age 9 (T1). In other words, mothers who maintained higher levels of PSE over time also had children who later engaged in lower age-18 RB behaviors during an important transitional phase characterized by increasing autonomy and detachment from family, such as late adolescence. Although, to our knowledge, this was the first study to examine how PSE’s developmental trends were associated with late adolescents’ behaviors, our results corroborated previous findings attesting to the key role of PSE for the health and well-being of children (see [21]). In particular, the results of this study supported the idea that mothers’ beliefs about the result of their parenting practices were a unique predictor of child behaviors, also during transitional periods that may impose barriers and setbacks and undermine motivation to act [13, 28].

However, these results were not replicated for fathers, which is consistent with the majority of previous studies that did not find support for the importance of fathers’ PSE in predicting child problematic behaviors [102, 103]. Although “new fathers” are more involved and present in the lives of children, it is also true that they often perceive their role as being mediated by mothers [104, 105], thus still perceiving the ability to successfully perform the parenting role as more maternal than paternal. Also, especially in the Italian context, mothers usually spend more time with their children than fathers, which may render child characteristics, especially behavioral problems, more of a challenge for mothers than fathers. Another explanation might be that mothers and fathers view various parenting tasks as being differentially important, and so they spend more effort in those tasks they deem as being especially pertinent [106]. For instance, fathers are historically and currently considered as having a salient role in providing financially for the family [107, 108]. It is possible then that observed differences in PSE between mothers and fathers might be a function of the instruments used to assess the construct. Future studies may help evidence aspects of parenting salient to men that fully represent which kind of fathering experiences are more relevant in predicting their children’s behaviors. The finding that RB behaviors were not affected by the initial levels of either mothers’ or fathers’ PSE was in line with existing developmental research suggesting that PSE varies across time and situational conditions rather than manifests uniformly across contexts [e.g., 13]. That is, independently of whether parents started with low or high levels of PSE, the amount of change that they experienced in their ability to influence the development of their children significantly contributed to predict late adolescents’ RB behaviors. Future research should explore different time periods to better understand the over time influences of PSE on child behaviors across developmental transitions.

In regards to covariates of our models (gender and SES), and in partial accordance with our hypothesis, we found that child gender was not significantly related with either mothers’ or fathers’ initial levels or changes in PSE over time, indicating large similarities in mothers’ and fathers’ levels of PSE with boys and girls. These results complemented the conclusions of two recent systematic reviews of PSE among parents of children and adolescents [23, 24], which found that levels of mothers’ and fathers’ PSE did not differ as a function of the gender of the child. It is possible that parents do not hold gender-differentiated perceptions of, and expectations for, their children’s competencies in specific areas (e.g., academic achievement or avoiding risky behaviors), and therefore they do not perceive their efficacy to differ between boys and girls. In line with this finding, there is evidence that gender-differentiated parenting practices have become less pronounced in many contemporary societies, with consistent differences in the treatment of boys and girls found only with regard to specific parenting dimensions (e.g., parental gender talk, parents’ toy, clothing, and chore choices for children, see [109]). Thus, mothers and fathers may differ in their gender-differentiated parenting beliefs and practices only with regard to very specific areas, which may not have been represented in our measure of PSE.

Moreover, child gender was not a significant predictor of adolescents’ RB behaviors. The zero-order correlations and the t-test indicated that at age 9 (T1), boys were more likely to engage in RB behaviors than girls. However, this gender difference was non-significant at age 18 (T7). Although we expected that males would have been overall higher than females in their levels of RB, this finding suggested that adolescent females engage in approximately similar levels of RB as their adolescent male counterparts, and is in accordance with previous findings on the diminishing sex-differences in adolescence [58, 110]. According to Moffitt [110], while, during childhood, males are more likely to show higher levels of RB behaviors than females, during adolescence males and females engage in similar levels of RB as a way try to demonstrate autonomy from parents, win affiliation with peers, and hasten social maturation. Future research should continue to examine differences in girls’ and boys’ RB behaviors to further clarify the heterogeneity in RB behaviors’ development, particularly across mid-to-late adolescence, when engagement in RB typically increases.

Regarding family SES, we found that higher initial levels of family SES were associated with lower initial levels of mothers’ and fathers’ PSE. This result was in line with our hypothesis and previous studies suggesting that PSE is stronger in settings that combine adversities and obstacles, such as financial strains [12, 105, 111]. In these stressful circumstances, parents with low-SES, compared with high-SES parents, cannot get by with a lower sense of PSE because they have greater stressors to cope with and fewer supplemental aids to guide their children’s development [13]. In these situations, parents may believe that more efforts are needed to help their children and so may set higher standards and greater aspirations for themselves as parents. However, we cannot rule out the possibility of potential differences in the interpretation of the PSE measure and in the relation between PSE and RB behaviors among low-and-high SES families. In order to gain a fuller picture, future research should test the invariance of the PSE scale among high-and-low SES parents and examine possible SES differences in the effect of PSE on adolescents’ RB behaviors by performing multigroup analyses.

In addition, and contrary to our expectations, SES positively predicted RB behaviors at age 18 (T7). Although previous studies found that youths from low-SES families reported higher levels of RB behaviors [77], it is also possible that adolescents coming from high-SES families have access to greater resources, freedom, and independence from others, which in turn give rise to decreased perceptions of risk associated with committing unethical acts and inattention to the consequences of one’s actions [112]. Another explanation may derive from the results of prior studies conducted by Luthar and colleagues [e.g., 113, 114] on *at-risk* children from high- and low-income families, which suggest that, compared to children from low-income families, affluent youths are at higher risk for serious adjustment problems because often pressed by their parents to excel at multiple academic and extracurricular pursuits and often are alone at home for several hours a week because of their many afterschool activities and the demands of affluent parents’ career obligations [114]. Thus, greater availability of resources and freedom, achievement pressure, and low closeness to parents may lead youths from high-SES families to engage in more RB behaviors to alleviate their personal distress and yearn for greater attention from parents.

### Strengths, limitations, and future research

To our knowledge, this study was the first to explore the developmental trends of mothers’ and fathers’ PSE and their association with adolescents’ RB behaviors over a span of seven waves. However, several limitations should be highlighted.

First, we did not investigate the influence of other parenting variables, such as parenting styles and/or co-parenting arrangements, that could have affected the development of mothers’ and fathers’ PSE and the relation between PSE and RB behaviors. Examining the role played by these variables (e.g., parental monitoring, psychological control, permissive parenting style, or spousal involvement and/or support) in influencing adolescents’ RB behaviors could help understanding the underlying mechanisms of the relation between PSE and RB behaviors. Second, in the present study, we did not consider important contextual (e.g., neighborhood or peer influences) and individual factors (e.g., parent and child temperament and personality characteristics) that may play a significant predictive role in shaping both the development of PSE and its association with adolescents’ RB behaviors.

Third, although our main interest was to investigate whether and how changes in PSE would predict late adolescents’ RB behaviors, we cannot exclude that levels of PSE could depend on previous levels of RB behaviors, and vice versa. Because previous studies suggested that PSE and children’s behaviors co-evolve in a transactional way, exerting a mutual influence on each other [29, 31], future studies may implement advanced longitudinal statistical techniques, such as the random intercept cross-lagged panel model [RI-CLPM; 115], to shed light on these complex dynamics, providing information regarding the relative importance of each construct across development. Finally, our results emerged from a convenience sample of Italian families. Further cross-cultural studies are needed to extend our findings.

### Conclusion

To our knowledge this was the first study tracing the developmental trends of mothers’ and fathers’ PSE over seven waves (representing children’s transition from late childhood to late adolescence; approximately from 9 to 18 years old) and examining whether and how initial levels and rates of change of mothers’ and fathers’ PSE predicted late adolescents’ rule-breaking (RB) behaviors. Overall, our findings provided useful insights in terms of both theoretical and practical implications.

From a theoretical perspective, our results added to the understanding of how PSE develops and changes over time across the transition to late adolescence, how these changes may be different between mothers and fathers, and how they relate to important child outcomes, such as adolescents’ RB behaviors. Overall, although mothers and fathers followed different developmental trends in their PSE over time, mothers who maintained a firm sense of PSE reduce the likelihood of negative adolescents’ adjustment. Thus, high levels of parents’ beliefs about their parenting capabilities, especially those of mothers, may function as a protective factor even during a period characterized by adolescents’ increased autonomy and detachment from the family.

From a practical perspective, our results suggested the importance of helping parents develop a strong sense of self-efficacy in supervising and supporting their adolescents’ behaviors and development. Intervention programs should be aimed at increasing PSE among mothers during the childhood years, as well as in the transition to late adolescence. For instance, parent training programs that provide active skills training (modelling, rehearsal and feedback; e.g., the Triple P-Positive Parenting Program and the Teen Triple P, [116, 117] and settings that are rich in experiential, vicarious, verbal, and affective stimuli, enhance family protective factors and reduce risk factors associated with child behavioral and emotional problems related to their developmental transitions. Moreover, interventions should aim at increasing fathers’ involvement in childrearing practices. With the considerably greater involvement of men in parenting, there is an increasing need for fathers to develop parenting skills that strengthen relationships and facilitate their children’s development [118]. Measures of PSE can serve as screening tools to help professionals involved in promoting positive parenting identify parents in need and calibrate their intervention actions to enhance feelings and beliefs of parenting competencies. When both mothers and fathers are included in prevention programs aimed at promoting positive parenting strategies, it becomes possible to investigate systemic family contributions that enhance feelings and beliefs of parents’ parenting competencies and reduce children’s problematic behaviors.

## Supporting information

S1 Fig Unconditional latent curve model for mothers. PSE-M = Mothers’ parental self-efficacy.
(TIF)

S2 Fig Unconditional latent curve model for fathers. PSE-F = Fathers’ parental self-efficacy.
(TIF)

S1 Table Sample sizes differentiated by site, time, and reporters. (DOCX)

S2 Table Measurement invariance of mothers’ parental self-efficacy across time (from T1 to T7). M = model; CFI = Comparative Fit Index. TLI = Robust Tucker-Lewis index; RMSEA = Root Mean Square Error of Approximation. CI = confidence interval. SRMR = Standardized Root Mean Square Residual.
(DOCX)

S3 Table Measurement invariance of fathers’ parental self-efficacy across time (from T1 to T7). M = model; CFI = Comparative Fit Index. TLI = Robust Tucker-Lewis index; RMSEA = Root Mean Square Error of Approximation. CI = confidence interval. SRMR = Standardized Root Mean Square Residual.
(DOCX)

S1 Data (XLSX)
