# Supplementary figures and images for: The developmental trends of parental self-efficacy and adolescents’ rule-breaking behaviors in the Italian context: A 7-wave latent growth curve study
Source: PLoS One. 2023 Nov 15;18(11):e0293911. doi: 10.1371/journal.pone.0293911 (PMC10651020; doi:10.1371/journal.pone.0293911)

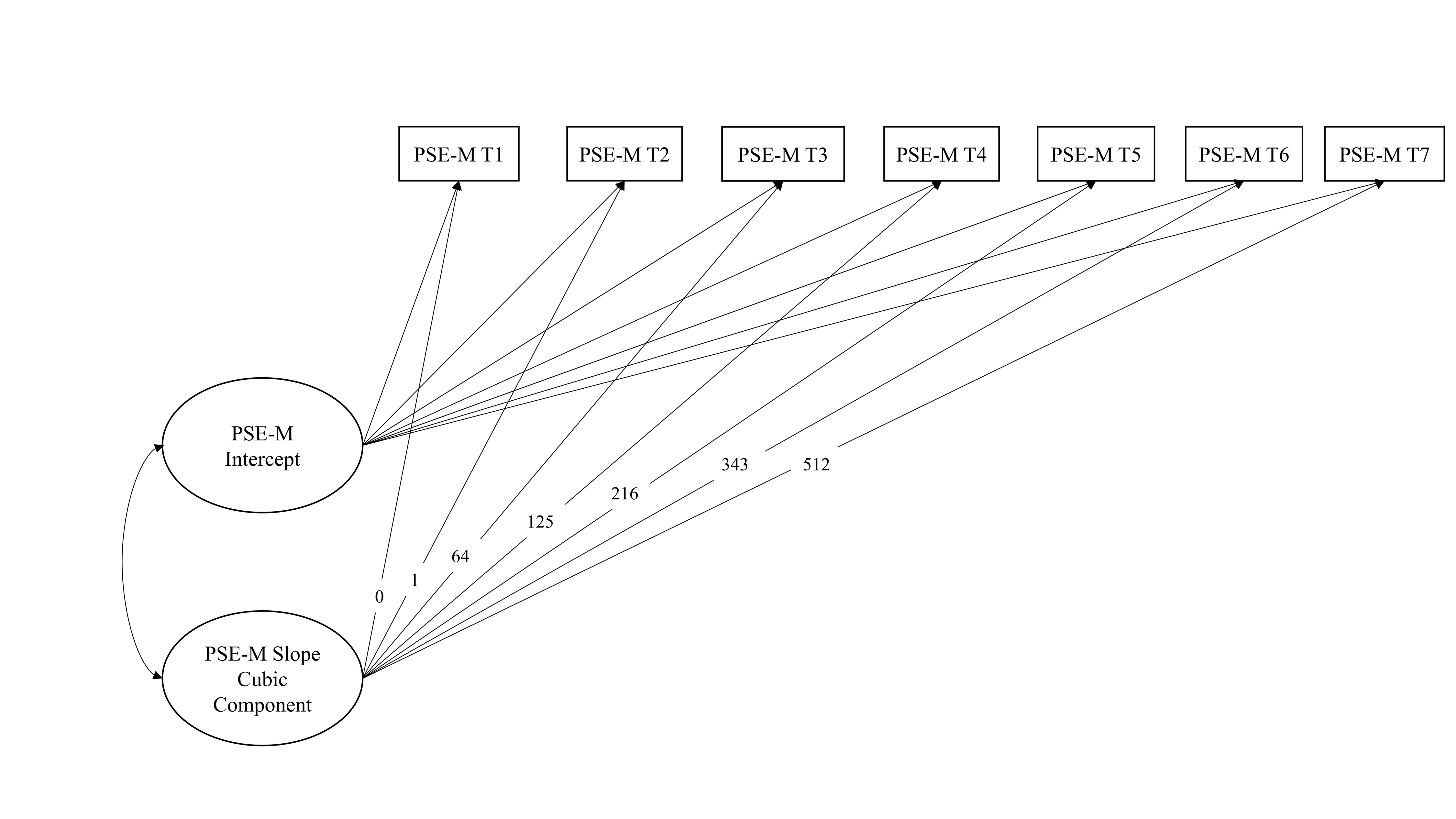

Supplement: S1 Fig — PSE-M = Mothers’ parental self-efficacy. (TIF) [file pone.0293911.s001.tif]

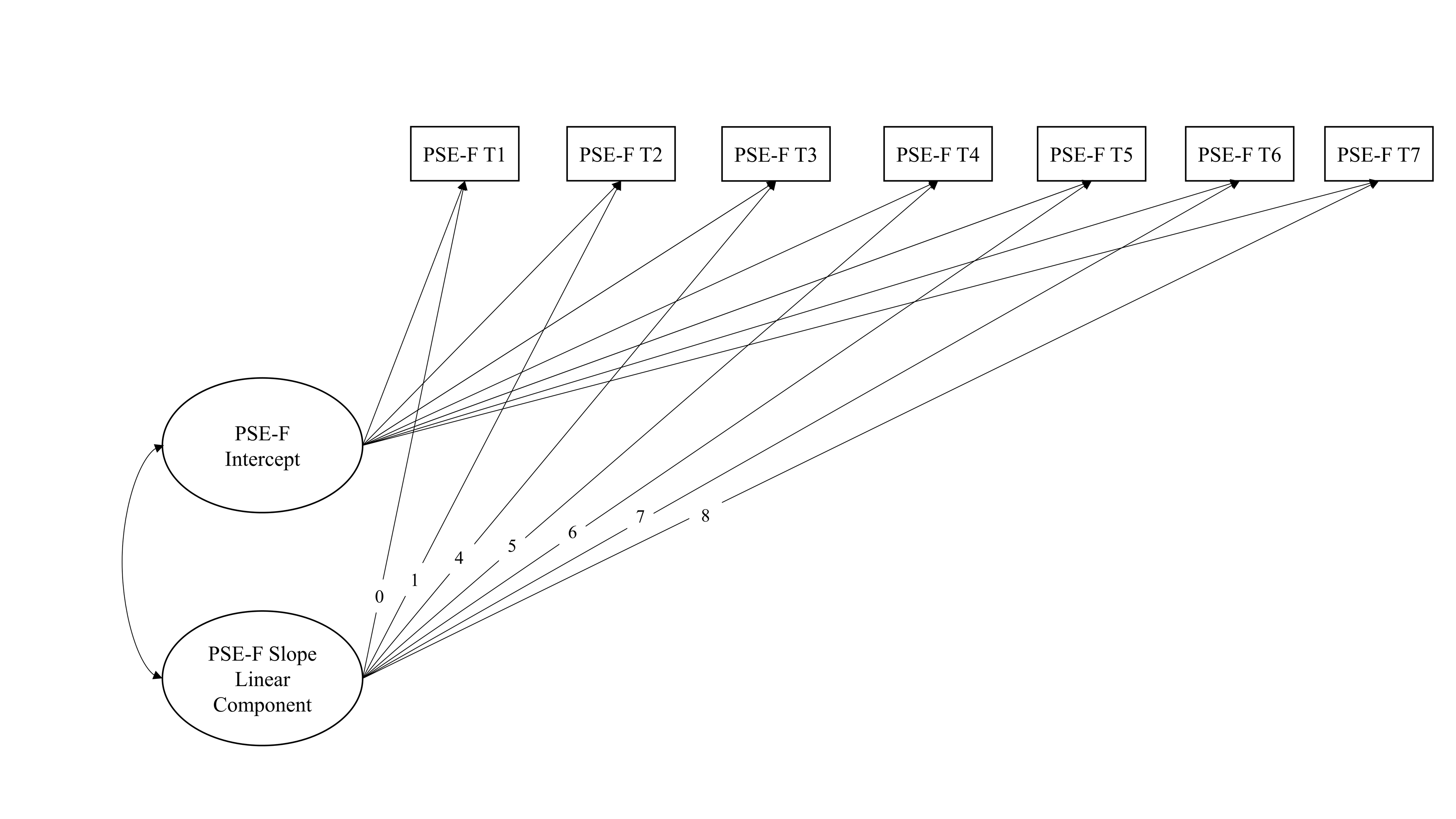

Supplement: S2 Fig — PSE-F = Fathers’ parental self-efficacy. (TIF) [file pone.0293911.s002.tif]
