# Supplementary material for: The developmental trends of parental self-efficacy and adolescents’ rule-breaking behaviors in the Italian context: A 7-wave latent growth curve study
Source: PLoS One. 2023 Nov 15;18(11):e0293911. doi: 10.1371/journal.pone.0293911 (PMC10651020; doi:10.1371/journal.pone.0293911)
Supplement: S1 Table — (DOCX) [file pone.0293911.s003.docx]

**S1 Table**

|  | Time 1 | Time 2 | Time 3 | Time 4 | Time 5 | Time 6 | Time 7 |
| --- | --- | --- | --- | --- | --- | --- | --- |
| Naples | Adolescents *n=*99  Mothers *n=*99; Fathers *n=*82 | Mothers *n=*95  Fathers *n*=83 | Mothers *n=*94  Fathers *n*=73 | Mothers *n=*92  Fathers *n*=73 | Mothers *n=*85  Fathers *n*=67 | Mothers *n=*84  Fathers *n*=66 | Adolescents *n=*77  Mothers *n=*74; Fathers *n=*58 |
| Rome | Adolescents *n=*99  Mothers *n=*101; Fathers *n=*76 | Mothers *n=*99  Fathers *n*=69 | Mothers *n=*98  Fathers *n*=75 | Mothers *n=*100  Fathers *n*=77 | Mothers *n=*102  Fathers *n*=79 | Mothers *n=*100  Fathers *n*=75 | Adolescents *n=*100  Mothers *n=*101; Fathers *n=*78 |
