# Supplementary material for: The developmental trends of parental self-efficacy and adolescents’ rule-breaking behaviors in the Italian context: A 7-wave latent growth curve study
Source: PLoS One. 2023 Nov 15;18(11):e0293911. doi: 10.1371/journal.pone.0293911 (PMC10651020; doi:10.1371/journal.pone.0293911)
Supplement: S2 Table — M = model; CFI = Comparative Fit Index. TLI = Robust Tucker-Lewis index; RMSEA = Root Mean Square Error of Approximation. CI = confidence interval. SRMR = Standardized Root Mean Square Residual. (DOCX) [file pone.0293911.s004.docx]

**S2 Table**

| Model | *χ2* | *df* | *p* | CFI | RMSEA (90%CI) | SRMR | MC | ΔCFI |
| --- | --- | --- | --- | --- | --- | --- | --- | --- |
| M1.Configural | 1061.757 | 738 | 0.000 | 0.902 | 0.046 (0.040 0.052) | 0.076 |  |  |
| M2.Metric | 1105.942 | 768 | 0.000 | 0.898 | 0.046 (0.040 0.052) | 0.086 | M2vsM1 | -0.004 |
| M3.Scalar | 1226.903 | 798 | 0.000 | 0.870 | 0.051 (0.045 0.056) | 0.089 | M3vsM2 | -0.028 |
|  | | | | | | | | |
