# Supplementary material for: The developmental trends of parental self-efficacy and adolescents’ rule-breaking behaviors in the Italian context: A 7-wave latent growth curve study
Source: PLoS One. 2023 Nov 15;18(11):e0293911. doi: 10.1371/journal.pone.0293911 (PMC10651020; doi:10.1371/journal.pone.0293911)
Supplement: S3 Table — M = model; CFI = Comparative Fit Index. TLI = Robust Tucker-Lewis index; RMSEA = Root Mean Square Error of Approximation. CI = confidence interval. SRMR = Standardized Root Mean Square Residual. (DOCX) [file pone.0293911.s005.docx]

**S3 Table**

| Model | *χ2* | *df* | *p* | CFI | RMSEA (90%CI) | SRMR | MC | ΔCFI |
| --- | --- | --- | --- | --- | --- | --- | --- | --- |
| M1. Configural | 1226.521 | 767 | 0.000 | 0.880 | 0.058 (0.052 0.064) | 0.072 |  |  |
| M2. Metric | 1285.970 | 797 | 0.000 | 0.872 | 0.059 (0.053 0.064) | 0.084 | M2vsM1 | -0.008 |
| M3. Scalar | 1423.850 | 827 | 0.000 | 0.844 | 0.063 (0.058 0.069) | 0.084 | M3vsM2 | -0.028 |
|  | | | | | | | | |
